# Dose-response relationship in digital psychological therapies for people with psychosis: a systematic review, meta-analysis, and meta-regression

**DOI:** 10.3389/fpsyt.2025.1621009

**Published:** 2025-09-26

**Authors:** Carolina Fialho, Jenny Yiend, Chloe Hampshire, Rayan Taher, Sukhi Shergill, Daniel Stahl

**Affiliations:** ^1^ Department of Psychosis Studies, Institute of Psychiatry, Psychology & Neuroscience, King’s College London, London, United Kingdom; ^2^ Department of Psychology, University of Bath, Bath, United Kingdom; ^3^ Kent and Medway Medical School, Canterbury, United Kingdom; ^4^ Department of Biostatistics and Health Informatics, Institute of Psychiatry, Psychology & Neuroscience, King’s College London, London, United Kingdom

**Keywords:** psychosis, dose-response, systematic review, meta-analysis, meta-regression

## Abstract

**Introduction:**

Recent digital technological advances have emerged with the aim of improving accessibility, engagement, and effectiveness of psychological interventions for psychosis. Systematic reviews have provided preliminary evidence that digital health technologies for psychosis may improve symptoms. However, little research has examined how treatment effect is related to dose of therapy. Thus, we planned to investigate the association between treatment outcome and different dose characteristics, such as session length, number of sessions and their frequency.

**Methods:**

This systematic review followed the PRISMA guidelines, including a risk of bias assessment utilizing the Cochrane Collaboration’s tool. Searches were completed in November 2023 using Embase, Ovid MEDLINE(R) and APA PsychInfo, and were limited to English language and peer-reviewed journal articles. Studies included any randomised controlled trial (including pilot/feasibility studies) in adults that reported a non-interventional control condition and included clinical symptom outcome measurement and dose information. Meta-analyses and meta-regressions were completed.

**Results:**

19 studies were included in this review. 14 studies included web, mobile or computer-based interventions, and 5 included virtual reality interventions. Digital interventions significantly improved clinical symptoms, with a small effect size (Cohen’s d = -0.14, p < 0.001, 95% CI [-0.23 to -0.05]). Although subgroup analyses were not significant, data patterns favoured interventions focusing on clinical outcomes over cognitive outcomes, and interventions that included therapist support, over those without. Due to the small overall effect size, we were not able to explore dose predictors.

**Discussion:**

This meta-analysis provided preliminary evidence that digital mental health interventions for psychosis are effective, even when not targeting symptoms directly. Despite exploring multiple dose characteristics, no significant dose-response relationship was found. Further research is needed to understand the role of dose in digital interventions for psychosis.

**Systematic review registration:**

https://www.crd.york.ac.uk/PROSPERO/view/CRD42023411836, identifier CRD42023411836.

## 1 Introduction

The current high demand for mental health services results in people with psychosis having limited or no access to psychological interventions. The National Institute for Health and Care Excellence (NICE) recognises that Digital Mental Health Interventions (DMHIs) have the potential to offer treatment to people who may otherwise not be able to access psychological interventions (1). Besides improving accessibility, these interventions also aim to improve engagement and effectiveness (2) and can include different technologies such as virtual reality, mobile apps, and computerised therapies. Systematic reviews have provided preliminary evidence that DMHIs for psychosis may improve symptoms (3, 4). For example, the Cognitive Bias Modification for paranoia (CBM-pa) computerised therapy reduced interpretation bias, paranoia, depression, and anxiety (5), while the virtual reality gameChange therapy significantly reduced agoraphobic avoidance and distress (6). Though literature on inpatient delivery is more limited, DMHIs have also shown benefits in these settings. For example, the virtual reality VRelax therapy significantly reduced stress, anxiety, and low mood (7), and the computerised cognitive remediation Drill Training programme reduced both positive and negative symptoms (8). Despite these developments being promising, little research has examined how treatment effect is related to dose of psychotherapy.

Dose of psychotherapy can be characterised by duration, frequency, and amount (9). Duration refers to the time period over which therapy is intended to be delivered (e.g. 10 weeks). Frequency refers to how often contact is intended to be made (e.g. twice per week). Amount refers to the length of each intended individual contact (e.g. 50 mins). The total dose can be identified by multiplying the duration, frequency, and amount (e.g. 10 x 2 x 50 = 1000 minutes of therapy); or more simply, by multiplying number of sessions and length of session (e.g. 20x50 mins= 1000min). Identifying the optimal dose of therapy has great practical implications, not only to inform treatment planning and maximise therapeutic benefits, but also to inform resource allocation.

One of the main models for understanding therapeutic change as a function of number of sessions attended is called the dose-response model. This model is characterized by a curvilinear relationship, suggesting that most of the symptomatic improvement is observed during the initial sessions of treatment and then generally plateaus after (10). Continuing a treatment which is no longer benefitting a patient may be considered an opportunity cost, since the patient could have accessed a more effective treatment sooner, and an inadequate use of limited resources. On the other hand, if the dose offered to a patient is lower than the dose required to achieve the plateau, this will translate into lower therapeutic benefits, and the patient may require an additional course of therapy in the future. Thus, it is of crucial importance to identify the optimal dose of therapies.

To the best of our knowledge, only a small number of studies have examined the dose-response effect for face-to-face and digital therapies for psychosis. For example, a meta-analysis on the dose-response relationship in music therapy identified a significant relationship: small effects were seen after 3 to 10 sessions, medium effects after 10-24 sessions, and large effects after 16-51 sessions (11). Another study investigated the minimal number of CBT-p sessions needed to achieve significant changes in clinical symptoms and reported that whilst a minimum of 15 sessions were required, the frequency of symptoms reached a minimum by session 25 (12). And finally, research on the dose-response of CBT in people who do not receive antipsychotic medication found that each CBT session attended reduced the primary outcome measure (PANSS score) (13). In digital therapies, the efficacy of CBM-pa computerised therapy was examined, with intervention effects evident following the third session (5). These studies highlight the impact that the number of sessions has on therapy outcomes.

However, when trying to understand the dose-response relationship, looking at the number of sessions is not enough. In order to have a comprehensive understanding of the relationship between dose and outcome, it is important to also have in consideration the length of each contact, frequency of contact, and duration of therapy. For example, different lengths of contact (e.g. 30-60 minutes), will lead to significantly different total contact time (in minutes). Similarly, different frequencies (e.g. weekly to monthly) will lead to different duration and intensity of therapy. There is limited understanding of the association between these components of dose and treatment effects, with several systematic reviews and meta-analyses having identified the need for future studies to investigate the optimal dose of interventions (4, 14, 15). Thus, in response to this gap in the literature, we conducted a meta-analysis and meta-regression analyses with the aim of answering the question: what dose of digital therapy is needed to improve clinical symptoms in people with psychosis across both outpatient and inpatient settings?

## 2 Methods

This review was conducted in accordance with the Preferred Reporting Items for Systematic Reviews and Meta-Analyses (PRISMA) guidelines (16). See **Supplementary File 1** for the completed PRISMA 2020 checklist.

### 2.1 Eligibility criteria

Inclusion and exclusion criteria were guided by the PICO (Population, Intervention, Comparison, and Outcome) framework. 1- Participants were predominantly adults with a psychotic disorder. 2- Interventions required the use of technology to deliver individualised therapy. Interventions delivered in group settings were only included if the intervention itself was individualized. Studies focusing on wellbeing apps not specifically for clinical use, or where the digital health technology is not an intervention, or where the digital component of the intervention is supplemental or minimal were excluded. 3- The comparison was a parallel control group (i.e. waitlist, treatment-as-usual). Studies where the only control group was an alternative treatment were excluded. 4- The outcomes included clinical symptoms measured by validated measures. Studies were included if they reported a change in clinical symptoms and the dose of therapy required to achieve this outcome. Types of study included randomised control trials (defined to include pilot, feasibility, or full-powered trials). Study protocols and any study that reports the same data as an already included study but in a different context were excluded.

### 2.2 Information sources

The search was completed in November 2023, on three databases (Embase, Ovid MEDLINE(R) and APA PsychInfo), and was limited to English language and peer-reviewed journal articles.

### 2.3 Search strategy

Please refer to **Supplementary File 2** for the search strategy carried out on the three databases.

### 2.4 Selection process

One author (CF) transferred the search findings to Rayyan (software that facilitates systematic reviews). Two authors (CF and CH) independently assessed titles and abstracts against the eligibility criteria. Following this, the two authors met to discuss the discrepancies and reach consensus. CF and CH then assessed the full texts of the included studies against the eligibility criteria. Upon its completion, CF and CH met again to discuss discrepancies. A senior author (JY) was consulted when consensus was not reached. The interrater reliability was calculated for both stages of screening, with a Cohen’s Kappa of 0.59 indicating moderate agreement during the abstract review, and a Kappa of 0.69 suggesting substantial agreement during the full-text assessment.

### 2.5 Data collection process

Two authors (CF and CH) piloted the data extraction Excel document. CF and CH independently extracted the data for each study and resolved discrepancies through discussion.

### 2.6 Data items

The following data was extracted:

- Study characteristics: authors, study title, year of publication, design (e.g. randomised control trial), setting (e.g. secondary care outpatient vs inpatient), type of control condition (e.g. treatment-as-usual), measure used to assess clinical symptoms (e.g. PANSS), and the level of the clinical outcome extracted (e.g. primary);
- Participant characteristics: diagnosis and key demographic data for the psychotherapy and control conditions (e.g. N, mean, standard deviation);
- Therapy characteristics: the digital health technology (e.g. virtual reality), psychotherapy type (e.g. targets clinical outcomes), treatment target (e.g. psychosis symptoms), and intensity of therapist support (e.g. therapist supports some or all elements of intervention);
- Dose characteristics: intended number of sessions, length of sessions (in minutes), total therapy time (number of sessions x length of sessions), frequency (number of sessions p/week), duration of treatment (in weeks), and average number of sessions attended;
- Clinical outcome data for both psychotherapy and control conditions at different time points: baseline and post-intervention.

When data from specific studies were missing or unclear, CF emailed the corresponding authors to request more information.

### 2.7 Study risk of bias assessment

The risk of bias was assessed using the Cochrane Collaboration’s ‘Risk of Bias’ assessment tool (17). Both ‘randomised parallel-group trials’ and ‘cluster-randomized parallel-group trials’ templates were used. Together, these assess bias due to the randomisation process, identification or recruitment of individual participants within clusters, deviations from intended interventions, missing outcome data, measurement of outcome, and selection of the reported result. Each category generated a level of risk: ‘low’, ‘some concerns’ or ‘high’, and contributed to an overall level of bias. Two reviewers independently assessed the risk of bias for all studies and resolved discrepancies in the overall level of bias through discussion. When disagreements on individual domains did not impact the overall bias assessment, reviewers chose the most conservative judgement or, if possible, a compromise between both evaluations (‘some concerns’).

### 2.8 Effect measures

For each outcome, we used standardised mean differences for the presentation of the results.

### 2.9 Synthesis methods

Our outcome for synthesis was clinical outcomes using any type of validated quantitative measure (clinician administered or self-report). Since the outcomes were continuous, we calculated Cohen’s d, defined as the mean difference between mean posttreatment and mean baseline measures divided by the pooled pretest standard deviation. We used standard deviation at baseline because change score standard deviations were not reported. This procedure results in the robust effect size reflecting the magnitude of change relative to the initial variability. The standard error for the effect sizes was calculated using a formula provided by Cooper and colleagues (18). Studies were weighted using an inverse variance method, meaning that studies with narrower confidence intervals, and larger precision, were given greater weight. The meta-analysis was done using a random-effects model, assuming that both within-group variability of scores and mean effect sizes are caused by differences between studies (between-study heterogeneity). Random-effects models incorporated between-group heterogeneity, resulting in estimates with wider confidence intervals than fixed-effects models, but more realistic in psychiatric studies due to the variety of case-mix, treatments, and settings between studies (19). A random-effects model was employed for the meta-regression analysis to assess the influence of moderators on the observed effect sizes.

The number of clinical outcomes reported varied between studies, with a total of 12 different outcomes reported, as well as separate PANSS subscale scores. To increase the number of studies, we calculated a standardized (or ‘normed’) data point using published population mean and standard deviation estimates (please refer to **Supplementary File 3** for the statistical norms used). Normative data are used to compare the characteristics of a group of people (or an individual) with data for the average person within a reference population.

For each study, we first calculated the change score between pre and baseline measure for treatment and control arm, respectively. We then calculated the normed mean change score by subtracting the published population mean from the mean change score of a study and divided it by the standard deviation of the population mean:


$$d_{norm}=\frac{\left(X-µ\right)}{sd}$$

where:

− X is the mean change score of treatment or control arm of a study,
− µ is the population norm mean, and.
− sd is the standard deviation of the normed population data.

The score d_norm_ represents the number of standard deviations and the reported study mean is from the population mean, making it easy to understand a study’s relative standing within a population. This method allows for comparisons across different measures with different scales. The final standardised effect size, Cohen’s d, is then calculated as the difference between the standardised mean change scores d_norm_ of treatment and control arm. The standard error of the effect size is calculated using the formula described previously (18) adapted to normative data and assuming a correlation between baseline and post measurements of r=0.5. For further details on the calculation of the Cohen’s d see **Supplementary File 4**.

Some studies provided data for more than one outcome, but the numbers were insufficient for a network meta-analysis. To avoid inflating the sample size when the same control group was used to calculate multiple effect sizes, we divided the control group’s sample size by the number of outcomes (17). As a sensitivity analysis, we then re-ran the meta-analyses using a multilevel approach, with study as a random effect, to account for potential dependencies of effect sizes within studies. For the meta-analysis, we required at least 5 studies to get a reliable estimate of combined effect sizes. For the meta-regression, it is advised to have more than 10 studies to ensure sufficient statistical power to assess the relationship between dose moderator and treatment effect.

We assessed the homogeneity of true effect sizes using Cochran’s Q test and quantified heterogeneity across studies with I², a sample size-independent measure of inconsistency (17). This allowed us to determine if there was significant between-study variance, indicating that a meta-regression could be useful to explore potential sources of heterogeneity.

Publication bias was assessed by i) visual inspection of funnel plots—a plot of study precision (1/standard error) against effect size, ii) Begg’s adjusted rank test and Egger’s test, and iii) the “trim and fill” method by Duval and Tweedie (20). The trim and fill method is a sensitivity analysis used to estimate and correct for missing studies likely due to publication bias by re-estimating the effect size. Another important bias is ‘poor trial quality bias,’ which can result in exaggerated effect sizes. Evidence for this bias includes a tendency for studies with small sample sizes to show large beneficial effects, which can also be detected in funnel plots.

Effect sizes were calculated to indicate the difference between the psychotherapy and the control group at post-test. The number of studies reporting treatment differences was insufficient to conduct reliable meta-analyses at other follow-up time points. We adopted an ‘intention-to-treat’ approach to the analyses by including all participants in the analysis as originally assigned in the study. Forest plots were used to visually display the effect sizes and confidence intervals for each study, alongside the overall effect size. To assess that results were not overly influenced by any single study, a leave-one-study-out sensitivity analysis was conducted, where each study is sequentially removed from the meta-analysis analysis to evaluate the consistency of the overall effect size.

In line with the variables prespecified in Prospero, we conducted five univariable metaregression analyses to examine the association between the effect size and the following dose characteristics:

1. Number of sessions (intended).
2. Length of sessions.
3. Total therapy time (length of sessions x number of sessions) (in minutes).
4. Frequency (number of sessions p/week).
5. Duration of treatment (in weeks).

We also conducted a separate analysis on the actual adherence rates by examining the association between the effect size and the average number of sessions attended. This was the only dose component that could be investigated, as other adherence-related components were not sufficiently reported. Finally, we conducted a subgroup meta-analysis to examine whether treatment effects differed by setting (e.g. secondary care outpatient vs inpatient).

## 3 Results

### 3.1 Study selection

**Figure 1:**
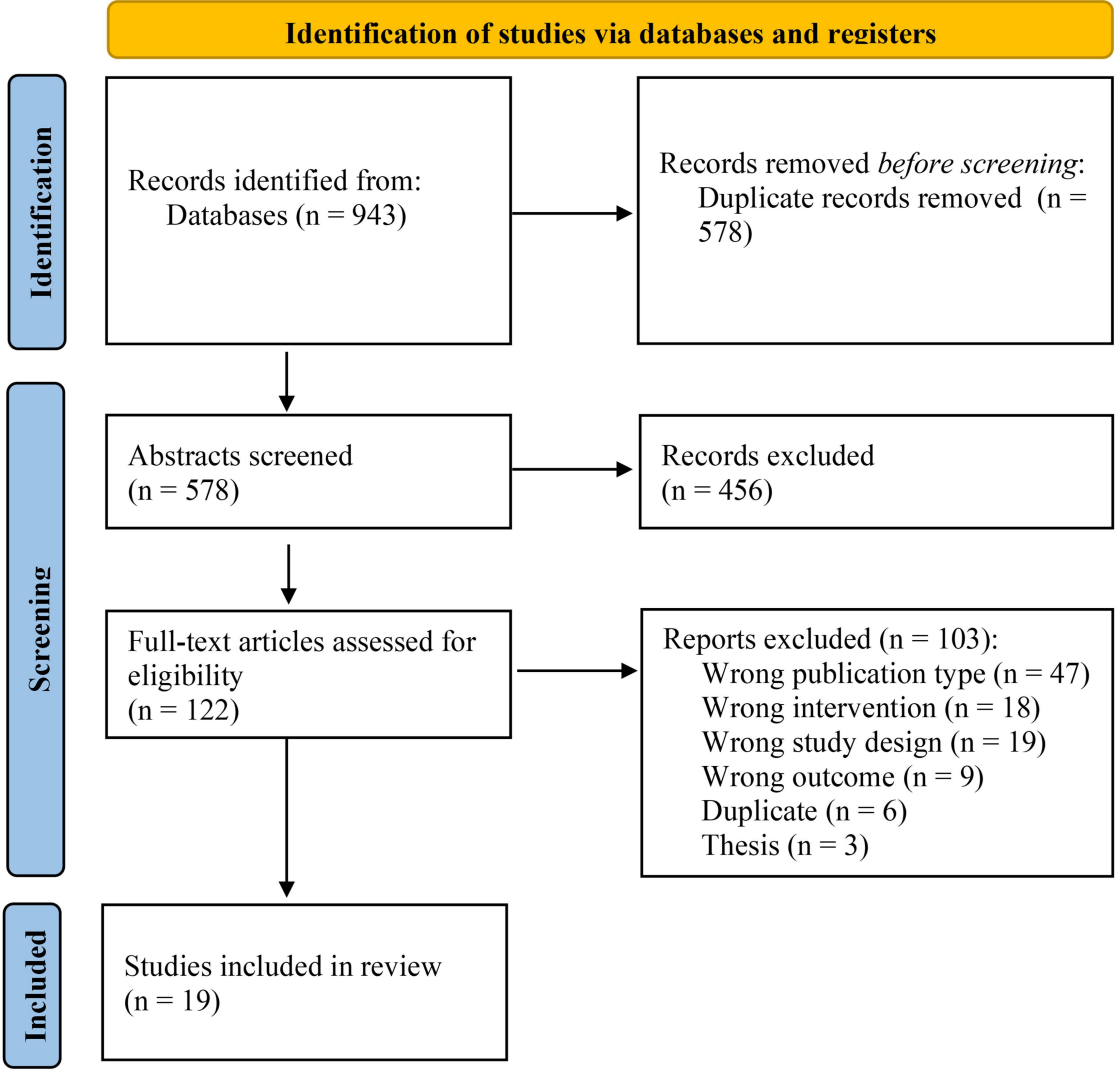
Flow diagram for the selection of studies.

The search strategy identified 578 records after removing duplicates. Titles and abstracts were screened against the inclusion and exclusion criteria, which led to the examination of 122 full texts against the eligibility criteria. 19 studies met full eligibility criteria and were included in the review. Please see **Figure 1** for the PRISMA flowchart of study selection, which includes further information on the reasons for study exclusion.

### 3.2 Study characteristics

The included studies were carried out in 11 countries, with the USA being the most prevalent (N=5). Publication dates ranged from 2002 to 2023. There were 14 RCTs and 5 pilot or feasibility studies. 15 studies were conducted in secondary care outpatient services, and 4 were in secondary care inpatient settings. 17 studies assessed digital interventions and 2 studies assessed blended interventions. While digital interventions were defined as therapies delivered primarily through digital platforms, blended interventions in this review combined face-to-face sessions with the use of a mobile app outside of clinician-led sessions. 14 studies included web, mobile or computer-based interventions, and 5 studies included virtual reality interventions. Most studies assessing virtual reality interventions employed seated or standing head-gaze interfaces to navigate the virtual environment, while one study required joystick-based locomotion.

11 of the studies had interventions that targeted cognitive outcomes, and 8 targeted clinical outcomes. 13 studies had therapists supporting some or all aspects of the intervention, whereas 6 studies did not have therapists providing support. Studies used 8 measures, including 15 subscales (PANSS was the most prominent; N=12).

The number of participants in the intervention groups ranged from 9 to 181 (mean =51.20; SD=50.05; Median=29.50), and in the control groups ranged from 7 to180 (M=49.55; SD=50.10; Median=28.00). The mean ages of the participants from the intervention groups ranged from 21.46 to 51.20 (M=39.90; SD=5.96) and from the control group from 22.3 to 48.8 (M=40.58, SD=5.99). The control condition in 13 studies consisted of TAU, and 6 were active non-interventional controls (e.g. computer games).

The number of sessions of the interventions ranged from 6 to 250 (M=31.60; SD=54.45; Median=16.00). In digital interventions, sessions lasted between 30 to 82.70 minutes (M=51.51; SD=14.29; Median=55.00). In blended interventions, sessions with the clinician lasted between 75 and 90 minutes (M=82.5, SD=7.5), and sessions via the mobile app lasted 1.5 minutes (1 study; mobile device prompted participants to complete thought-challenging surveys three times a day). The derived dose (number of sessions x length of sessions) varied from 3 to 80 hours (M=17.45; SD=17.23; Median=13.21). The average number of sessions attended varied from 2.90 to 172.06 (M=25.98; SD=38.85; Median=16.00). The total duration of therapy lasted from 4 to 24 weeks (M=10.01; SD=4.77; Median=10.00). The number of sessions per week ranged from 0.25 to 20.83 (M=3.19; SD=4.43; Median=1.84). The broad variations in dose parameters were due to the diverse types of intervention and technologies used. The blended therapy, augmented by a mobile app, permitted shorter, more frequent, and more numerous prescribed sessions, due to therapists not being involved in the digital sessions. In contrast, digital interventions delivered via virtual reality or computerised platforms, which required therapist involvement, led to fewer, longer and less frequent prescribed sessions. Please refer to **Supplementary File 5** for the full study characteristics.

### 3.3 Risk of bias in studies

Of the 19 studies included, 16 were judged to have some risk of bias (84.21%) and 3 had low risk of bias (15.79%). Deviations from the intended intervention and the selection of the reported result were the main sources of bias. Please see **Supplementary File 6** for more details on the RoB2 findings.

### 3.4 Results of individual studies

Please see **Supplementary File 7** for the summary statistics for the intervention and control groups across the included studies.

### 3.5 Results of syntheses

#### 3.5.1 Overall effects

**Figure 2:**
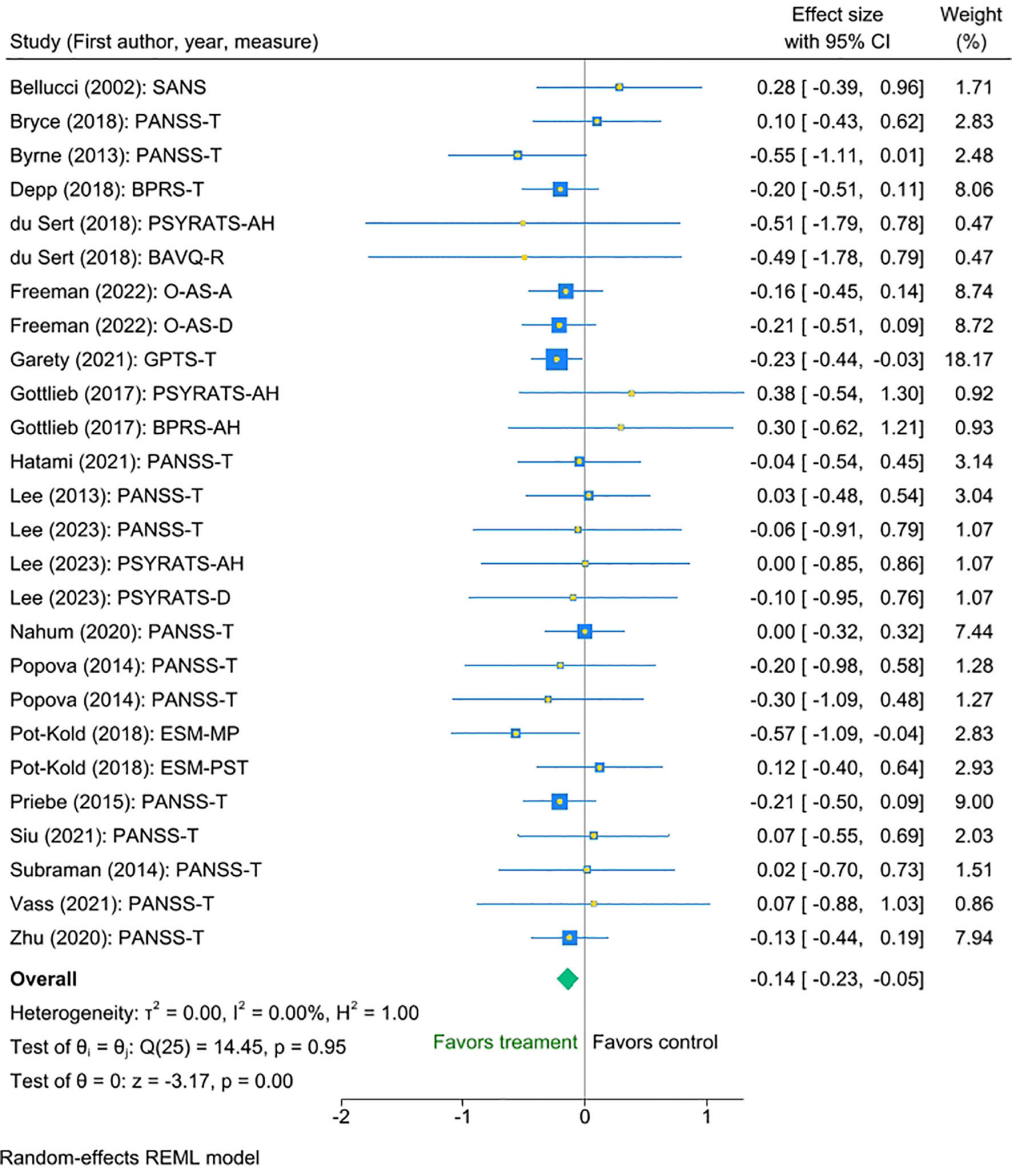
Meta-analysis of the effect of digital interventions on clinical symptoms: overall treatment effect.

The multilevel meta-analysis showed that digital interventions significantly improved clinical symptoms post-intervention for the treatment condition compared with the control condition, with a small effect size (Cohen’s d = -0.14, SE = 0.05, Z = -3.17, p < 0.001, 95% CI [-0.23 to -0.05]; **Figure 2**). There was no significant heterogeneity between the studies [I² = 0.00%, Q_M = χ² (25) = 14.45, p = 0.95.

#### 3.5.2 Association of effect size with dose characteristics

We examined the association between the effect size and each dose component (please refer to **Table 1** for full details). There was no statistically significant effect of any dose components (length of sessions, number of sessions, total therapy time, frequency, duration of treatment, and average number of sessions attended) on the outcome.

**Table 1:** Standardized regression coefficients of dose characteristics of digital psychotherapies: univariable meta-regression analyses.

Dose characteristics	Number of observations, n	Cohen’s *d* [95% Confidence Interval]	Standard error	z	P>|z|
Number of sessions	26	-0.0001 [-0.0014, 0.0013]	0.0007	-0.1200	0.9040
Length of sessions	24	0.0040 [-0.0036, 0.0116]	0.0039	1.0300	0.3020
Total therapy time (length of sessions x number of sessions) (in minutes)	26	0.0001 [0.0000, 0.0002]	0.0001	1.1000	0.2730
Frequency (number of sessions p/week)	26	-0.0015 [-0.0180, 0.0150]	0.0084	-0.1800	0.8590
Duration of treatment (in weeks)	26	-0.0004 [-0.0181, 0.0173]	0.0090	-0.0400	0.9650
Average number of sessions attended	20	-0.0004 [-0.0025, 0.0017]	0.0011	-0.3500	0.7270

#### 3.5.3 Effect of psychotherapy type, intensity of therapist support, and setting

**Figure 3:**
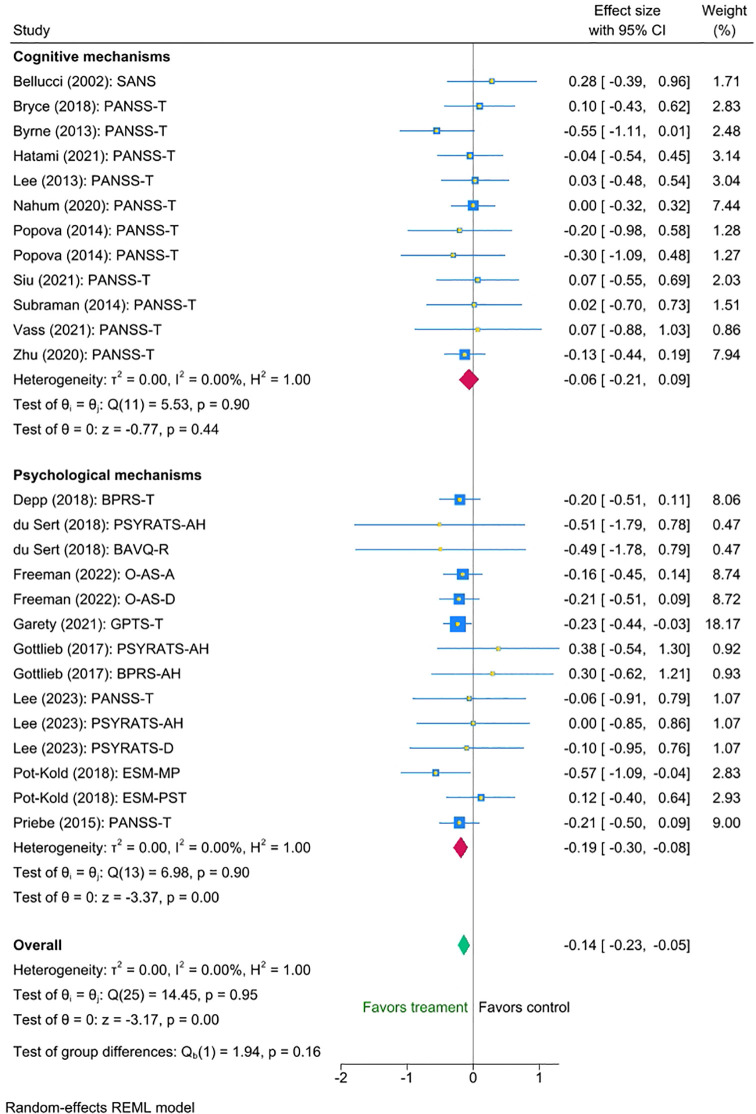
Meta-analysis of the effect of digital interventions on clinical symptoms: by psychotherapy type.

Three factors of potential interest - psychotherapy type, intensity of therapist support, and setting - had sufficient variability of data to investigate their impact on the interventions’ efficacy. Thus, we conducted three multi-level meta-analyses to investigate them. We found no statistically significant differences between the efficacy of interventions targeting cognitive and clinical outcomes (mean difference -0.13, 95% CI [-0.32 to 0.05), p = 0.16). Nonetheless, exploratory inspection of the subgroups revealed that interventions directly targeting clinical outcomes were associated with a small statistically significant reduction on symptoms (Cohen’s d = -0.19, SE=0.055, Z = -3.37, p < 0.001, 95% CI [-0.30 to -0.08], N=14), whereas interventions targeting cognitive outcomes showed a smaller non-significant effect (Cohen’s d = -0.06, SE=0.074, Z = -0.77, p = 0.44, 95% CI [-0.21 to 0.09, N=12]). Please refer to **Figure 3** for the forest plot.

**Figure 4:**
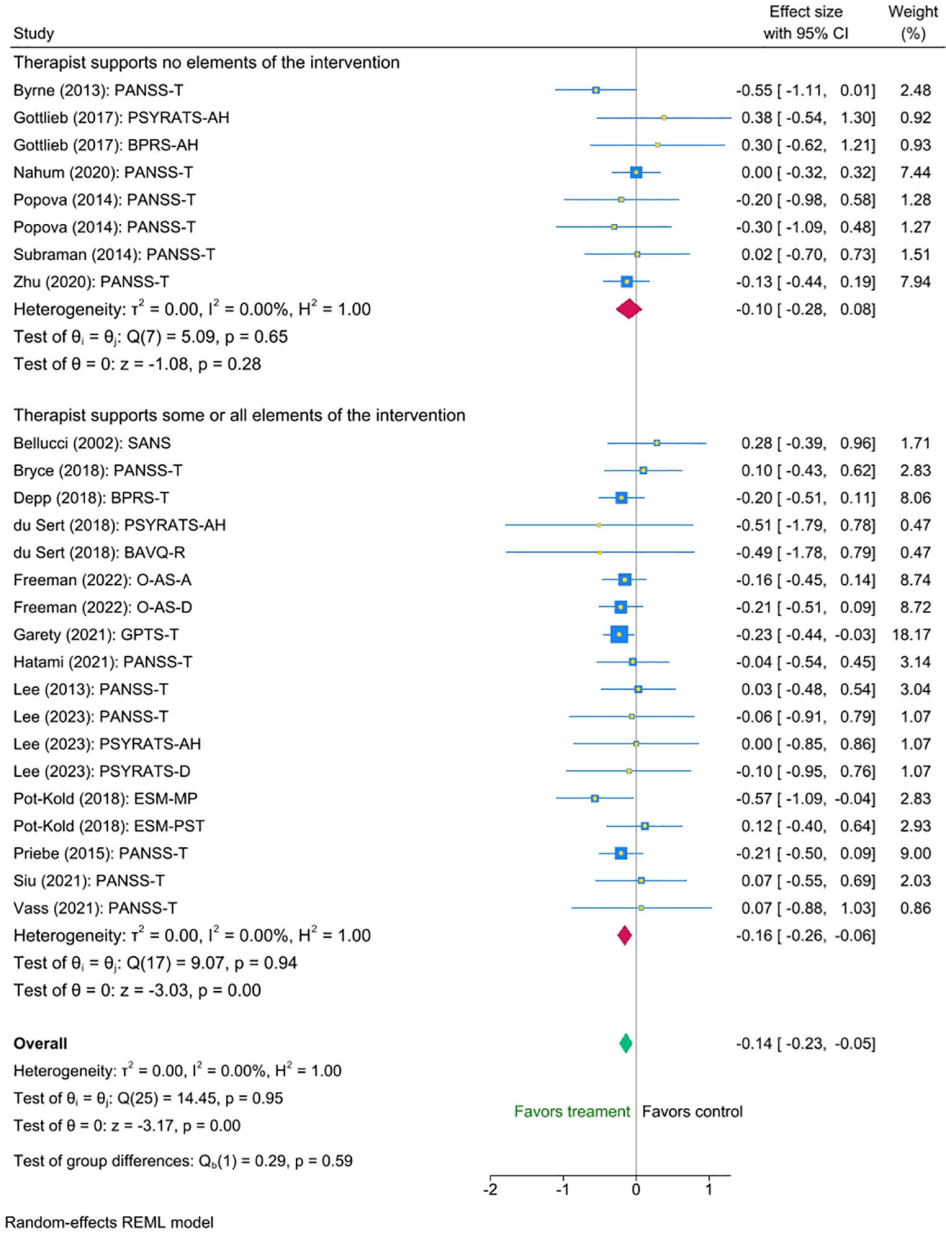
Meta-analysis of the effect of digital interventions on clinical symptoms: by intensity of therapist support.

Similarly, we found no statistically significant difference between the groups with and without therapist support (mean difference = -0.06 (95% CI [-0.26 to 0.15], p = 0.59). Nonetheless, exploratory inspection of the subgroups showed that studies in which therapists supported some or all aspects of the intervention displayed a small statistically significant reduction in symptoms (Cohen’s d = -0.16, SE=0.051,Z = -3.03, p < 0.001, 95% CI [-0.26 to -0.06]), N=18, whereas interventions without support showed a smaller non-significant effect (Cohen’s d = -0.10, Se=0.091, Z = -1.08, p = 0.28, 95% CI [-0.28 to 0.08], N=8). Please refer to **Figure 4** for the forest plot.

**Figure 5:**
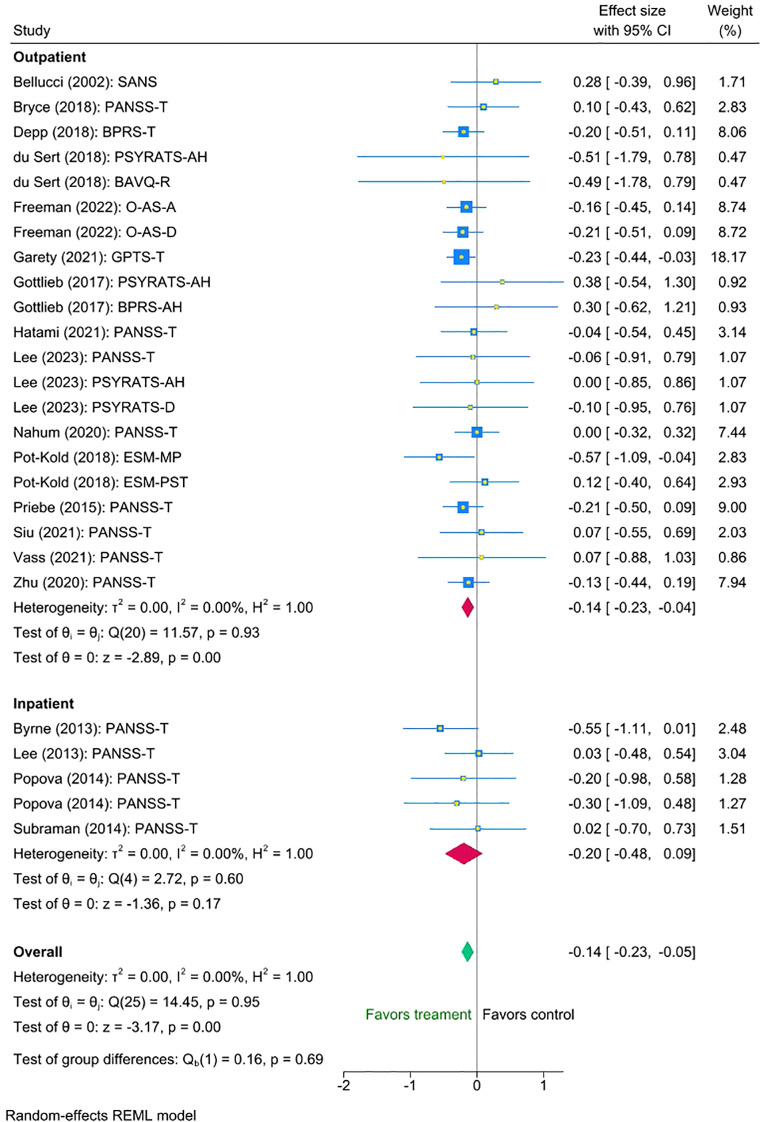
Meta-analysis of the effect of digital interventions on clinical symptoms: by setting.

Finally, we found no statistically significant difference between the outpatient and inpatient group studies (mean difference = -0.06 (95% CI [-0.36 to 0.23], p = 0.69). Exploratory inspection of the subgroups showed that studies with outpatients displayed a small statistically significant reduction in symptoms (Cohen’s d = -0.14, SE = 0.047, Z = -2.89, p < 0.001, 95% CI [-0.23 to -0.04], N = 21), whereas the small number of interventions with inpatient groups suggested a somewhat larger, though non-significant effect (Cohen’s d = -0.20, SE = 0.142, Z = -1.36, p = 0.60, 95% CI [-0.48 to 0.09], N = 5). Please refer to **Figure 5** for the forest plot.

### 3.6 Reporting biases

**Figure 6:**
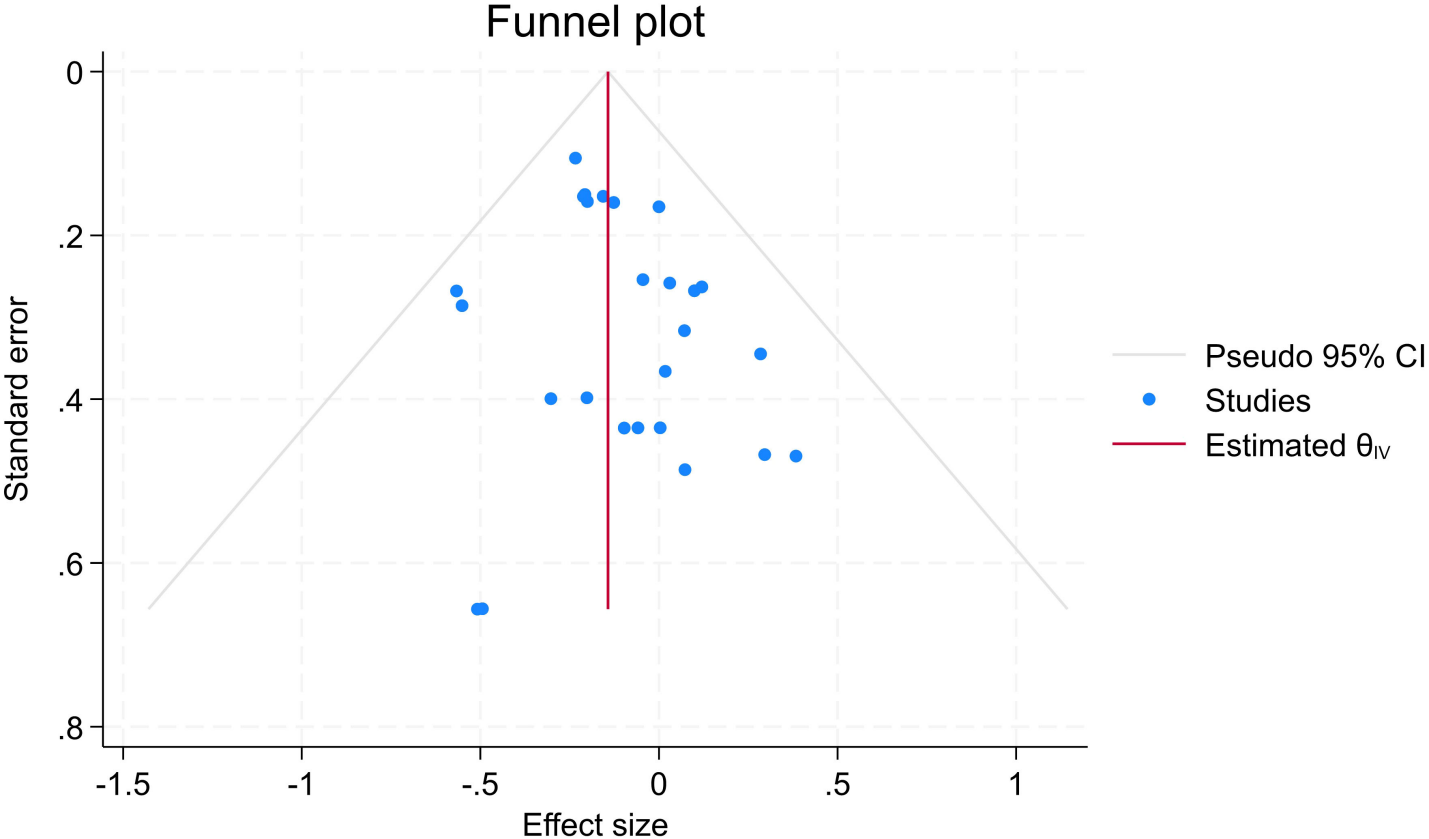
Funnel plot for assessing publication bias.

The funnel plot showed asymmetry, with effect sizes concentrated on the left (negative values), indicating possible treatment effects (symptom reduction) rather than publication bias (see **Figure 6** for further details). This is supported by a trim-and-fill analysis, which estimated four missing studies and imputed the potential missing values, resulting in a change in effect size from d = -0.143 to -0.168 (95% CI: -0.254 to -0.083), supporting the validity of the observed treatment effect. Begg’s and Egger tests for small study effects also did not suggest publication bias (p=0.33 and p=028, respectively).

### 3.7 Sensitivity analysis

A leave-one-study-out sensitivity analysis did not reveal any influential studies. Effect sizes, after omitting each study, ranged from -0.122 to -0.155 and remained significant throughout.

## 4 Discussion

This meta-analysis and meta-regression aimed to investigate the effect of digital mental health interventions for psychosis on core clinical symptoms and its association with different dose characteristics. For the purposes of this review, clinical symptoms encompass positive symptoms (e.g., hallucinations, delusions), negative symptoms (e.g., social withdrawal, blunted affect), and general psychiatric symptoms. Our results suggested that digital mental health interventions for psychosis significantly improved the severity of clinical symptoms, with a small effect size. Although differences between groups were not significant, we also found data patterns favoring symptom reduction when interventions focused on clinical outcomes rather than cognitive ones, and when therapists were involved in supporting the digital intervention.

There was no statistically significant effect on clinical symptoms of any of the dose components we investigated (length of sessions, number of sessions, total therapy time, frequency, duration of treatment, and average number of sessions attended). Despite this suggesting that therapeutic dose does not significantly impact the effectiveness of digital therapies for psychosis, these results should be interpreted with caution. The sample sizes within individual studies were small and there were relatively few studies included in the review, meaning true effects may have been missed. However, the noticeably small confidence intervals involved in the dose analyses suggest that even with larger samples and more studies, finding significant effects of dose would be unlikely. One key reason for this may be that we are comparing interventions that exhibit considerable heterogeneity, not only in modality (e.g. web-based vs immersive virtual reality, the latter requiring more advanced cognitive and spatial skills) but also in their therapeutic content. Moreover, differences in patient populations (such as diagnosis, symptom severity, and engagement levels) could also further obscure potential dose-response relationships.

These findings also raise other interesting questions for the field of digital therapeutics in this patient group. Might digital therapeutic effects operate in an ‘all or none’ fashion with limited incremental gains to be made beyond a minimum dose? Or perhaps traditional conceptualisations of dose make less sense in the context of digital interventions where individual usage evidently ranges widely (from as little as 1.5 minutes to as much as 82 minutes in one sitting, according to our findings). Emerging frameworks in digital health research propose that ‘dose’ may be better understood as a combination of different dimensions beyond quantity of use, including: content received, user actions within the platform, and behavior changes targeted outside the intervention (21). In addition, both the prescribed (intended) and actual (enacted) doses in each domain may be relevant for clinical outcomes. Adopting this multi-dimensional approach could provide better insights into how doses of digital interventions influence outcomes in psychosis.

Building on the promising results of a previous meta-analysis by Clarke and colleagues (3), our review aimed to broaden the time frame covered and investigate dose components as predictors. Although we conducted a more updated evaluation of DMHIs, we reached similar findings: DMHIs are promising, however further and larger studies are needed to establish their effectiveness. The current review identified a small overall effect for the impact of digital interventions on clinical symptoms (Cohen’s d = -0.14). While this effect size may appear modest, it may still have practical relevance in clinical practice, due to the scalability and accessibility of digital interventions, and their potential to help address service constraints. Moreover, research has shown that even a small shift in mean scores on mental health measures can translate into substantial levels when scaled up to a population level, which means that ‘small’ effect sizes can in some contexts be large and impactful (22). Thus, even seemingly modest improvements can be meaningful for some individuals with psychosis, especially when other treatment options are limited or unavailable.

Another possible explanation for the small effect size observed is that many of the included interventions focused on cognitive impairments, rather than being designed to address clinical symptoms directly. Although outcomes for cognitive versus psychological interventions did not significantly differ, exploratory inspection of the subgroups suggested that psychological interventions had the largest effects on symptoms. A similar pattern was also highlighted in the meta-analysis by Clarke and colleagues, where web-based programs and phone apps directly targeting psychotic symptoms showed the most promise, with only one study reporting significant results for cognitive remediation. These findings highlight the importance of developing digital interventions that directly address clinical symptoms, as well as the need for more tailored and personalized approaches, which may ultimately produce larger effects.

Many interventions included therapist support for some or all aspects of their implementation. Although the overall difference between interventions with and without therapist support was not statistically significant, exploratory subgroup analyses suggested better outcomes with therapist involvement. These findings indicate that adding therapist support may be beneficial, although the evidence remains inconclusive. Moreover, the level of therapist involvement varied across studies, from technical support to full delivery of sessions, making it unclear which level is more effective. This may also depend on individual characteristics: low motivation, more severe paranoia and cognitive difficulties, older age, and limited digital access have been shown to make it challenging for some individuals with psychosis to engage in digital interventions (23, 24). This highlights the need for research to investigate personalized approaches that match the level of support to patient needs. Nonetheless, therapist-supported interventions can still help address the global shortage of mental health professionals, as they allow for support to be effectively provided by less specialized staff. For example, ‘supportive accountability’ is a model of delivery that suggests any human support can effectively increase adherence by creating a level of accountability between the user and a trusted, supportive ‘coach’ (25). This aligns with the guidelines described in the Early Value Assessment by NICE (1), which recommends both self-guided and guided interventions.

Differences in treatment settings may have influenced the findings, as inpatient and outpatient environments differ substantially in clinical and cognitive symptom severity, as well as functional capacity. Although subgroup analyses found no statistically significant difference between settings, there were too few inpatient studies to draw firm conclusions on this point. However, exploratory analyses suggested a trend towards larger effects for inpatient studies. If this is confirmed in future research, it could indicate that aspects of the inpatient environment contribute to better outcomes. Another interesting finding is that only one of the five interventions delivered in inpatient settings included therapist support, suggesting that, despite differences in symptom severity and functional capacity, patients in inpatient settings may not require more therapist involvement than those in outpatient interventions.

Even though adherence plays a critical role in determining the effectiveness of digital interventions, it was insufficiently reported in the included studies, limiting our ability to analyse its impact on outcomes. While low adherence may dilute the efficacy of interventions, high adherence likely maximises it. Research should routinely collect and report standardised adherence metrics such as number of sessions completed and actual frequency and length of sessions. Other useful usage metrics include: number of logins, time spent using the intervention, and module completion rates (26, 27). Using digital analytics and collecting real-time engagement data (i.e. ecological momentary assessment) can clarify engagement patterns and support the integration of adherence as a key outcome.

This meta-analysis and meta-regression has several limitations. First, the absence of an unbiased rater at abstract screening may have introduced bias. Future reviews should involve a third reviewer with a PhD or equivalent at all article selection phases to enhance scientific rigor.

Second, the inclusion of a small number of studies, with mostly small sample sizes, may have impacted the reliability of the results. It certainly impacted our ability to effectively investigate the association between the different components of dose and outcomes. However, the small confidence intervals identified suggest that even with a larger sample size, no significant dosing effects would likely be found. In addition, the limited number of studies and small sample sizes may have also prevented us from identifying heterogeneity in the analyses, despite studies exhibiting considerable actual heterogeneity. There were differences in: study design (RCT vs feasibility or pilot), setting (secondary care outpatient vs inpatient), digital health technology (web, mobile or computer-based ​vs virtual reality), psychotherapy type (targeting cognitive vs clinical outcomes), intensity of therapist support (therapist supports some or all aspects of intervention vs therapist supports no aspects of intervention), and clinical measures used. Such clinical and methodological heterogeneity complicated direct comparison between studies, diluted the overall effect size of our analysis, and introduced potential for biases (e.g. pilot studies are particularly prone to selection bias, as they often recruit small and non-representative samples). As a result, the pooled findings should be interpreted with caution, as they reflect a synthesis across highly varied interventions and study designs.

Finally, including studies with only non-intervention control conditions aimed to improve the analysis of the benefit of specific interventions, but it led to the exclusion of many studies investigating the efficacy of digital interventions. These limitations highlight an important gap in the literature, which warrants additional studies to determine the optimal dosing components of digital interventions for psychosis.

Future research should prioritise investigating not only the efficacy, but also the dosing characteristics of digital interventions for psychosis. These studies should assess how different dosing schedules affect treatment outcomes, for example by changing the frequency of sessions while keeping the total number constant, or by systematically varying the ‘derived dose’ (i.e. total duration of time engaged in therapy) envelope. To ensure the robustness of these findings, studies should employ larger sample sizes and investigate meaningful differences between cognitive and psychological interventions. In line with findings elsewhere, research should also investigate the possibility of personalising dose based on clinical needs and personal preferences, an approach that digital technologies are very well positioned to facilitate (28). And lastly, future reviews should focus on identifying optimal doses for specific digital interventions and include studies with both intervention and non-intervention control conditions. While waiting for the digital therapeutic literature to grow, it would be beneficial to interrogate dose within face-to-face therapies. This could be achieved through reviewing the large face-to-face therapy literature, using a similar quantitative approach to that adopted here. Although this has been done for depression (29), there is still a need for this specifically in the area of traditional psychotherapies for psychosis. Additionally, qualitative research to understand clinicians’ and users’ views about therapy dose are likely to become increasingly important, especially in the digital domain; indeed, some work in this field has already commenced (28, 30). Robust qualitative understanding of the digital therapeutic space is crucial for the success of digital implementation, as scepticism and negativity regarding interventions have been reported as barriers to user engagement and clinical integration (31, 32). Furthermore, some features of established therapies may usefully inform the design and implementation of digital interventions and improve their acceptability in clinical settings. Finally, involving users in the developmental phase, including dose considerations and delivery schedules, ensures that interventions are usable, engaging and practical, all of which are crucial for successful adherence (33).

## 5 Conclusions

Overall, our meta-analysis provided preliminary evidence that digital mental health interventions for psychosis are helpful in treating these conditions, even when not targeting clinical symptoms directly. However, the early stage of this literature did not allow us to draw conclusions on their optimal dosage. Despite these limitations, our findings highlight the potential for digital interventions to address mental health service constraints, both in the UK and worldwide. Further high-quality research focused on dose-response of digital interventions is needed. This will facilitate the development of more effective, scalable, and personalised treatments.

## Data Availability

The original contributions presented in the study are included in the article/**Supplementary Material**. Further inquiries can be directed to the corresponding author.
