# Supplementary material for: Dose-response relationship in digital psychological therapies for people with psychosis: a systematic review, meta-analysis, and meta-regression
Source: Front Psychiatry. 2025 Sep 26;16:1621009. doi: 10.3389/fpsyt.2025.1621009 (PMC12512042; doi:10.3389/fpsyt.2025.1621009)
Supplement: Supplementary file 1 [file DataSheet1.zip › Supplementary File 2.DOCX]

**Supplementary File 2 - Search Strategy**

| 1 | "session*".ab,ti. |
| --- | --- |
| 2 | "phone app*".ab,ti. |
| 3 | avatar therapy.ab,ti. |
| 4 | virtual reality.ab,ti. |
| 5 | VR.ab,ti. |
| 6 | "computer-assisted therap*".ab,ti. |
| 7 | "digital health intervention*".ab,ti. |
| 8 | eHealth.ab,ti. |
| 9 | mHealth.ab,ti. |
| 10 | "smartphone*".ab,ti. |
| 11 | "mobile app*".ab,ti. |
| 12 | internet.ab,ti. |
| 13 | online.ab,ti. |
| 14 | computer.ab,ti. |
| 15 | web-based.ab,ti. |
| 16 | digital mental health.ab,ti. |
| 17 | website.ab,ti. |
| 18 | telehealth.ab,ti. |
| 19 | "mobile*".ab,ti. |
| 20 | "delusion*".ab,ti. |
| 21 | "hallucination*".ab,ti. |
| 22 | "paranoi*".ab,ti. |
| 23 | psychosis.ab,ti. |
| 24 | schizophrenia.ab,ti. |
| 25 | positive symptoms.ab,ti. |
| 26 | negative symptoms.ab,ti. |
| 27 | 2 or 3 or 4 or 5 or 6 or 7 or 8 or 9 or 10 or 11 or 12 or 13 or 14 or 15 or 16 or 17 or 18 or 19 |
| 28 | 20 or 21 or 22 or 23 or 24 or 25 or 26 |
| 29 | 1 and 27 and 28 |
| 30 | remove duplicates from 29 |
