# Supplementary material for: Dose-response relationship in digital psychological therapies for people with psychosis: a systematic review, meta-analysis, and meta-regression
Source: Front Psychiatry. 2025 Sep 26;16:1621009. doi: 10.3389/fpsyt.2025.1621009 (PMC12512042; doi:10.3389/fpsyt.2025.1621009)
Supplement: Supplementary file 1 [file DataSheet1.zip › Supplementary File 3.DOCX]

**Supplementary File 3 - Statistical norms**

| Name of measure | M | SD | Author |
| --- | --- | --- | --- |
| PANSS Positive | 18.20 | 6.08 | Kay et al. (1987) (1) |
| PANSS Negative | 21.01 | 6.17 | Kay et al. (1987) (1) |
| PANSS General | 37.54 | 9.49 | Kay et al. (1987) (1) |
| PSYRATS – Auditory Hallucinations | 14.4 | 14.6 | Steel et al. (2007) (2) |
| PSYRATS – Delusions | 13.5 | 7.1 | Steel et al. (2007) (2) |
| ESM – Momentary Paranoia | 2.15 | 0.9 | Collip et al. (2011) (3) |
| ESM – Perceived Social Threat | 2.1 | 0.75 | Collip et al. (2011) (3) |
| SANS | 29.3 | 17.6 | Czobor et al. (1991) (4) |
| BPRS-24 – Hallucinations | 1.60 | 1.25 | Bajraktarov et al. (2023) (5) |
| BAVQ-R Omnipotence | 11.1 | 4.5 | Chadwick et al. (2000) (6) |
| BAVQ-R Malevolence | 10.1 | 5.5 | Chadwick et al. (2000) (6) |
| O-AS – Avoidance | 3.34 | 2.56 | Lambe et al. (2023) (7) |
| O-AS - Distress | 52.3 | 15.6 | Lambe et al. (2023) (7) |
| GPTS – Social Reference | 46.4 | 16.4 | Green et al. (2008) (8) |
| GPTS - Persecution | 55.4 | 15.7 | Green et al. (2008) (8) |

**References**

1. Kay SR, Flszbeln A, Qpjer LA. The Positive and Negative Syndrome Scale (PANSS) for Schizophrenia. Schizophr Bull [Internet]. 1987;13(2). Available from: https://academic.oup.com/schizophreniabulletin/article/13/2/261/1919795

2. Steel C, Garety PA, Freeman D, Craig E, Kuipers E, Bebbington P, et al. The multidimensional measurement of the positive symptoms of psychosis. Int J Methods Psychiatr Res. 2007;16(2):88–96.

3. Collip D, Oorschot M, Thewissen V, Van Os J, Bentall R, Myin-Germeys I. Social world interactions: How company connects to paranoia. Psychol Med. 2011 May;41(5):911–21.

4. Czobor P, Bitter L, Volavka J. Relationship Between the Brief Psychiatric Rating Scale and the Scale for the Assessment of Negative Symptoms: A Study of Their Correlation and Redundancy. Psychiatry Res. 1991;36:129–39.

5. Bajraktarov S, Blazhevska Stoilkovska B, Russo M, Repišti S, Maric NP, Dzubur Kulenovic A, et al. Factor structure of the brief psychiatric rating scale-expanded among outpatients with psychotic disorders in five Southeast European countries: evidence for five factors. Front Psychiatry. 2023;14.

6. Chadwick P, Lees S, Birchwood M. The revised beliefs about voices questionnaire (BAVQ-R). British Journal of Psychiatry. 2000;177(SEPT):229–32.

7. Lambe S, Bird JC, Loe BS, Rosebrock L, Kabir T, Petit A, et al. The Oxford Agoraphobic Avoidance Scale. Psychol Med. 2023 Mar 23;53(4):1233–43.

8. Green CEL, Freeman D, Kuipers E, Bebbington P, Fowler D, Dunn G, et al. Measuring ideas of persecution and social reference: The Green et al. Paranoid Thought Scales (GPTS). Psychol Med. 2008 Jan;38(1):101–11.
