# Supplementary material for: Dose-response relationship in digital psychological therapies for people with psychosis: a systematic review, meta-analysis, and meta-regression
Source: Front Psychiatry. 2025 Sep 26;16:1621009. doi: 10.3389/fpsyt.2025.1621009 (PMC12512042; doi:10.3389/fpsyt.2025.1621009)
Supplement: Supplementary file 1 [file DataSheet1.zip › Supplementary File 4.DOCX]

**Supplementary File 4 - Calculation of Cohen's d based on standardized change scores**

- **Step 1: Calculate the Change Scores**

For the treatment group, the change score is calculated as:

ΔT = μT_post - μT_baseline

For the control group, the change score is calculated as:

ΔC = μC_post - μC_baseline

Where:

μT_post is the mean of the treatment group at the post-measure.
μT_baseline is the mean of the treatment group at baseline.
μC_post is the mean of the control group at the post-measure.
μC_baseline is the mean of the control group at baseline.

- **Step 2: Standardize the Change Scores**

The standardized change scores (d_norm_)are calculated using the population standard deviation (σ_pop):

For the treatment group:

d_norm_ _ΔT = (ΔT / σ_pop)

For the control group:

d_norm_ _ΔC = (ΔC / σ_pop)

- **Step 3: Calculate the Difference Between the Standardized Change Scores**

The difference between the standardized change scores is:

d = d_norm_ _ΔT - d_norm_ _ΔC

Substituting the standardized change scores:

d = (μT_post - μT_baseline) / σ_pop - (μC_post - μC_baseline) / σ_pop

- **Step 4: Simplify the Formula**

Now, let's simplify the expression:

d = (μT_post - μT_baseline - μC_post + μC_baseline) / σ_pop

This can be further expanded and rearranged as:

d = (μT_post - μT_baseline - μC_post + μC_baseline) / σ_pop

This simplification shows that Cohen's d reflects the difference in changes between the two groups, normalized by the population's variability. The population mean cancels out during the subtraction of baseline and post scores, confirming the validity of the final formula.

- **Step 5: Include the Standard Deviation (SD) in the Final Formula**

The standard deviation of the difference between the two standardized change scores, assuming a correlation (r) of 0.5, is calculated as:

SD_d = sqrt[(SD_ΔT^2 / nT · σ_pop^2) + (SD_ΔC^2 / nC · σ_pop^2)]

Thus, the final Cohen's d with its standard deviation can be represented as:

d ± SD_d
