# Supplementary material for: Dose-response relationship in digital psychological therapies for people with psychosis: a systematic review, meta-analysis, and meta-regression
Source: Front Psychiatry. 2025 Sep 26;16:1621009. doi: 10.3389/fpsyt.2025.1621009 (PMC12512042; doi:10.3389/fpsyt.2025.1621009)
Supplement: Supplementary file 1 [file DataSheet1.zip › Supplementary File 5.DOCX]

**Supplementary File 5 - Characteristics of the included studies and outcomes**

Table 1. Selected characteristics of the included studies

| **Author** | **Country** | **Des** | **Setting** | **DHT** | **Psy type** | **N_Psy_** | **Age_Psy_ M(SD)** | **N male_Psi_** | **Con type** | **N_Con_** | **Age_Con_ M(SD)** | **N** **male _Con_** | **N_Sessions_** | **Session length (in min)** | **Derived dose** | **N_Sessions attended_** | **Durat (in weeks)** | **Sess/wk** | **Ther involv** | **Measures** |
| --- | --- | --- | --- | --- | --- | --- | --- | --- | --- | --- | --- | --- | --- | --- | --- | --- | --- | --- | --- | --- |
| Bellucci et al., 2002 | USA | RCT | Sec Comm | Web, mob, or comp​ | Cog out | 17 | 42.00 (Missing) | 8 | TAU | 17 | 42.00 (Missing) | 8 | 16 | 30.00 | 480.00 | 16.00 | 8.00 | 2.00 | Some or all​ | SANS |
| Bryce et al., 2018 | Australia | RCT | Sec Comm | Web, mob, or comp​ | Cog out | 29 | 40.34 (9.62) | 19 | Act | 27 | 41.78 (9.35) | 20 | 20 | 60.00 | 1200.00 | 12.95 | 10.00 | 2.00 | Some or all​ | PANSS |
| Byrne et al., 2013 | China | Pil & Feas | Inpat | Web, mob, or comp​ | Cog out | 27 | 45.15 (9.81) | 14 | TAU | 24 | 46.04 (8.68) | 17 | 12 | 45.00 | 575.10 | 12.78 | 6.00 | 2.50 | None​ | PANNS |
| Depp et al., 2018 | USA | RCT | Sec Comm | Web, mob, or comp​ | Psycho out | 77 | 51.20 (11.50) | 42 | TAU | 83 | 48.10 (11.70) | 42 | Therapist = 1  DHT= 249  Total = 250 | Therapist = 90  DHT = 1.5 | Therapist = 90  DHT = 373.5  Total dose = 463.5 | Therapist = 1  DHT = 68.7%  Total = 172.6 | 12.00 | Therapist = 1   DHT = 20.75  Total = 20.83 | Some or all​ | BPRS-T |
| Du Sert et al., 2018 | Canada | Pil & Feas | Sec Comm | VR​ | Psycho out | 15 | 42.90 (12.40) | 10 | TAU | 7 | 42.90 (12.40) | 5 | 7 | 45.00 | 315.00 | 6.20 | 7.00 | 1.00 | Some or all​ | PSYRATS-AH   BAVQ-R |
| Freeman et al., 2022 | UK | RCT | Sec Comm | VR​ | Psycho out | 174 | 36.60 (12.80) | 116 | TAU | 172 | 37.80 (12.20) | 115 | 6 | 30.00 | 180.00 | Missing | 6.00 | 1.00 | Some or all​ | O-AS Avoidance and Distress |
| Garety et al., 2021 | UK | RCT | Sec Comm | Web, mob, or comp​ | Psycho out | 181 | 43.10 (11.70) | 132 | TAU | 180 | 42.20 (11.60) | 120 | Web app = 8  Mob  App = 1 | Web app: Mid point = 75.00  Mob App: Missing | Web app = 600  Mob App: Missing  Total dose = 600 | Web app = 6.80   Mob App = 18.32   Total = 25.12 | 12.00 | Web app = 0.67  Mob App = 0.08  Total = 0.75 | Some or all​ | GPTS Total Score |
| Gottlieb et al., 2017 | USA | RCT | Sec Comm | Web, mob, or comp​ | Psycho out | 19 | 43.79 (13.16) | 9 | TAU | 18 | 40.28 (11.69) | 14 | 11 | 82.70 | 909.70 | 11 | 11.00 | 1.00 | None​ | PSYRATS-AH   BPRS- AH |
| Hatami et al., 2021 | Iran | RCT | Sec Comm | Web, mob, or comp​ | Cog out | 31 | 35.90 (8.5) | N/A | TAU | 31 | 38.90 (9.50) | N/A | 10 | 67.50 | 675.00 | Completed 10 (n=27)  Completed <10 (n=4)  Total= 9.35 | 4.00 | 2.5 | Some or all​ | PANSS |
| Lee et al., 2013 | Korea | RCT | Inpat | Web, mob, or comp​ | Cog out | 30 | 43.53 (4.87) | 16 | TAU | 30 | 43.46 (3.53) | 17 | 20 | 60.00 | 1200.00 | 25 completed 20 or more sessions | 12.00 | 1.67 | Some or all​ | PANSS |
| Lee et al., 2023 | Korea | Pil & Feas | Sec Comm | VR​ | Psycho out | 35 | 32.94 (11.00) | 15 | Act | 29 | 31.32 (10.37) | 13 | 8 | 30.00 | 240.00 | Missing | 8.00 | 1.00 | Some or all​ | PSYRATS-D  PSYRATS-AH   PANSS |
| Nahum et al., 2020 | USA | RCT | Sec Comm | Web, mob, or comp​ | Cog out | 76 | 42.50 (13.90) | 53 | Act | 71 | 43.27 (11.50) | 49 | 40 | 42.00 | 1680.00 | 27.20 | 10.00 | 4 | None​ | PANSS |
| Popova et al., 2014 | Germany | RCT | Inpat | Web, mob, or comp​ | Cog out | FAT = 19  CE = 19 | FAT = 39.6 (7.9)  CE = 36.0 (8.5) | FAT = 11  CE = 12 | TAU | 19 | 35.90 (10.60) | 15 | 20 | 60.00 | 1200.00 | 20.00 | 4.00 | 5.00 | None​ | PANSS |
| Pot-Kolder et al., 2018 | Netherlands | RCT | Sec Comm | VR​ | Psycho out | 58 | 36.50 (10.0) | 40 | TAU | 58 | 39.50 (10.00) | 42 | 16 | 60.00 | 960.00 | 13.31 | 10.00 | 1.60 | Some or all​ | ESM - Momentary paranoia   ESM - Perceived social threat |
| Priebe et al., 2015 | UK | RCT | Sec Comm | Web, mob, or comp​ | Psycho out | 94 | 41.50 (10.70) | 66 | Act | 85 | 41.70 (9.30) | 57 | 6 | 40.00 | 240.00 | 1.45 | 24.00 | 0.25 | Some or all​ | PANSS |
| Siu et al., 2021 | Hong Kong/China | Pil & Feas | Sec Comm | Web, mob, or comp​ | Cog out | 20 | 21.46 (3.67) | 8 | TAU | 20 | 22.3 (3.36) | 10 | 22 | 60.00 | 1320.00 | 22.00 | 15.1 | 1.46 | Some or all​ | PANSS |
| Subramaniam et al., 2014 | USA | RCT | Inpat | Web, mob, or comp​ | Cog out | 16 | 40.69 (12.70) | 12 | Act | 14 | 41.21 (9.48) | 10 | 80 | 60.00 | 4800.00 | Missing | 16.00 | 5.00 | None​ | PANSS |
| Vass et al., 2021 | Hungary | Pil & Feas | Sec Comm | VR​ | Cog out | 9 | 38.6 (13.49) | 5 | Act | 8 | 48.8 (8.87) | 3 | 9 | 50.00 | 450.00 | 9.00 | 9 | 1.00 | Some or all​ | PANSS |
| Zhu et al., 2020 | China | RCT | Sec Comm | Web, mob, or comp​ | Cog out | 78 | 43.7 (9.24) | 42 | TAU | 79 | 43.65 (8.64) | 43 | 50 | 45.00 | 2250.00 | 43.17 | 12 | 4.17 | None​ | PANSS |

**Abbreviations**

Act – active control; Age_Con_ M(SD) - Age Mean and Standard Deviation in the Control condition; Age_Psy_ M(SD) – Age Mean and Standard Deviation in the Psychotherapy condition; BAVQ-R - Beliefs about voices questionnaire – revised; BPRS-T – Brief Psychiatric Rating Scale - Total; BPRS- AH - Brief Psychiatric Rating Scale Auditory Hallucinations; Cog out – Cognitive outcomes; Con type – Control type; Derived dose – number of sessions x length of sessions; Des – Study Design; DHT – Digital Health Technology; Durat (in weeks) – duration of therapy (in weeks); ESM – Experience Sampling Method; GPTS – Green Paranoid Thoughts Scale; Inpat – Secondary Care Inpatient; N male_Con_ – Number of males in Psychotherapy condition; N male_Psi_ –Number of males in Psychotherapy condition; N_Con_ – N in the Control condition; None – therapist does not support any elements of the intervention; N_Psy_ – N in the Psychotherapy condition; N_Sessions_ – Number of sessions (intended); N_Sessions attended_ – average number of sessions attended; O-AS Avoidance and Distress – Oxford Agoraphobic Scale Avoidance and Distress; PANSS - Positive and Negative Syndrome Scale; Pil & Feas – Pilot & Feasibility; Psy type – Psychotherapy type; Psycho out – Psychological outcomes; PSYRATS-AH - Psychotic Symptom Rating Scales Auditory Hallucinations; PSYRATS-D – Psychotic Symptom Rating Scales Delusions; RCT – Randomised Controlled Trial; SANS – Scale for the Assessment of Negative Symptoms; Sec Comm - Secondary Care Community; Sess/wk – number of sessions per week (total number of sessions divided by the duration of therapy); Session length (in min) – Session length in minutes; Some or all – therapist supports some or all elements of the intervention; TAU – Treatment-as-usual; Ther involve – amount of therapist involvement; VR – Virtual Reality; Web, mob, or comp – Web, mobile, or computer-based.

Table 2. Mean and SD of the PANNS subscales and PANNS Total without imputations

| Author | Psychotherapy Group  Baseline  Mean (SD) | Psychotherapy Group Post-Intervention Mean (SD) | Control Group  Baseline Mean (SD) | Control Group Post-Intervention Mean (SD) |
| --- | --- | --- | --- | --- |
| Bryce et al., 2018 | Total: 61.82 (3.20) | Total: 60.96 (SE: 3.2) | Total: 60.29 (SE: 3.28) | Total: 57.66 (SE:3.33) |
| Byrne et al., 2013 | Positive: 17.55 (8.97)  Negative: 19.88 (6.87)  General: 33.00 (11.16) | Positive: 13.64 (7.34)  Negative: 16.14 (3.89)  General: 28.43 (8.41) | Positive: 13.59 (4.87)  Negative: 20.04 (4.42)  General: 31.04 (4.68) | Positive: 13.43 (4.44)   Negative: 19.43 (6.17)  General: 29.43 (4.24) |
| Hatami et al., 2021 | Total: 45.20 (3.80) | Total: 42.70 (5.40) | Total: 45.90 (3.80) | Total: 44.20 (5.10) |
| Lee et al., 2013 | Positive: 15.21 (3.65)  Negative: 15.43 (4.24)  General: 33.29 (6.89) | Positive: 15.64 (3.63)  Negative: 15.58 (4.65)  General: 33.74 (8.26) | Positive: 15.20 (3.65)  Negative: 15.69 (4.69)  General: 33.22 (6.78) | Positive: 15.74 (4.72)  Negative: 15.74 (4.75)  General: 33.12 (7.65) |
| Lee et al., 2023 | Total: 48.68 (11.44) | Total: 43.03 (9.81) | Total: 47.93 (9.55) | Total: 43.33 (10.16) |
| Nahum et al., 2020 | Total: 62.16 (15.02) | Total: 56.10 (14.41) | Total: 61.27 (15.55) | Total: 55.21 (12.94) |
| Popova et al., 2014 | *1 - Facial affect recognition training*  Positive: 16.10 (5.20)  Negative: 19.20 (6.60)  General: 36.90 (8.40)  *2- Cognitive Exercises*  Positive: 15.60 (5.20)  Negative: 18.00 (6.50)  General: 35.40 (5.50) | *1- Facial affect recognition training*  Positive: 14.40 (4.6)  Negative: 17.90 (6.2)  General: 33.20 (8.2)  *2 - Cognitive Exercises*  Positive: 12.60 (4.2)  Negative: 17.40 (6.8)  General: 30.50 (7.1) | Positive: 14.70 (4.90)  Negative: 19.60 (6.10)  General: 35.10 (9.00) | Positive: 13.60 (6.00)  Negative: 18.30 (6.20)  General: 34.30 (9.90) |
| Priebe et al., 2015 | Positive: 14.80 (5.70)  Negative: 17.10 (6.40)  General: 32.90 (8.30) | Positive: 13.20 (5.20)  Negative: 15.10 (5.80)  General: 28.00 (9.20) | Positive: 15.10 (6.40)  Negative: 18.00 (7.80)  General: 34.60 (10.10) | Positive: 14.40 (5.70)  Negative: 15.70 (6.10)  General: 32.80 (8.90) |
| Siu et al., 2021 | Positive: 8.17 (2.01)  Negative: 9.44 (3.05) | Positive: 8.22 (2.76)  Negative: 7.39 (1.04) | Positive:9.45 (4.11)  Negative: 8.70 (2.41) | Positive: 7.70 (1.49)  Negative: 7.60 (1.27) |
| Subramaniam et al., 2014 | Positive: 2.92 (1.02)  Negative: 2.25 (0.97) | Positive: 2.61 (1.00)  Negative: 2.43 (0.81) | Positive: 2.81 (1.15)  Negative: 2.26 (0.86) | Positive: 2.56 (1.18)  Negative: 2.35 (0.61) |
| Vass et al., 2021 | Positive: .10.10 (2.85)  Negative: 17.80 (6.38)  Cognitive: 13.80 (4.22)  Activity/excitement: 4.80 (1.21)  Affective: 9.1 (2.85) | Positive: 16.4 (21.27)  Negative: 15 (4.16)  Cognitive: 11.20 (4.80)  Activity/excitement: 6.00 (2.70)  Affective: 9.7 (5.93) | Positive: 11.20 (3.32)  Negative:17.50 (4.89)  Cognitive: 15.70 (4.55)  Activity/excitement: 6.30 (2.06)  Affective: 9.2 (4.97) | Positive: 10.20 (3.53)  Negative: 19.1 (5.58)  Cognitive: 16.20 (4.55)  Activity/excitement: 6.60 (2.26)  Affective: 9.2 (4.13) |
| Zhu et al., 2020 | Total: 48.65 (10.19) | Total: 43.14 (8.78) | Total: 50.57 (12.5) | Total: 47.32 (15.01) |

**Assumptions made during the analyses**

We made three assumptions in the analyses. For Hatami et al. (2021), we calculated the average number of sessions completed by assuming that people who “completed less than 10 sessions” attended 5 sessions. For Lee et al. (2013), we assumed that people who attended “20 or more sessions” attended 20 sessions. Finally, for Garety et al. (2021), we did not know the length of sessions on the mobile app and, thus, did not include it in the derived dose.
