# Supplementary material for: Dose-response relationship in digital psychological therapies for people with psychosis: a systematic review, meta-analysis, and meta-regression
Source: Front Psychiatry. 2025 Sep 26;16:1621009. doi: 10.3389/fpsyt.2025.1621009 (PMC12512042; doi:10.3389/fpsyt.2025.1621009)
Supplement: Supplementary file 1 [file DataSheet1.zip › Supplementary File 6.DOCX]

**Supplementary File 6 -** **Risk of bias summary per domain**

|  | **Study ID** | **Domain 1a** | **Domain 1b** | **Domain 2** | **Domain 3** | **Domain 4** | **Domain 5** | **Overall risk of bias** |  | **Risk of Bias**  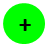Low risk  Some concerns |
| --- | --- | --- | --- | --- | --- | --- | --- | --- | --- | --- |
| 1 | Bellucci et al., 2002 |  | NA |  |  |  |  |  |  |  |
| 2 | Bryce et al., 2018 |  | NA |  |  |  |  |  |  |  |
| 3 | Byrne et al., 2013 |  | NA |  |  |  |  |  |  |  |
| 4 | Depp et al., 2018 |  | NA |  |  |  |  |  |  |  |
| 5 | Du Sert et al., 2018 |  | NA |  |  |  |  |  |  |  |
| 6 | Freeman et al., 2022 |  | NA |  |  |  |  |  |  |  |
| 7 | Garety et al., 2021 |  | NA |  |  |  |  |  |  |  |
| 8 | Gottlieb et al., 2018 |  | NA |  |  |  |  |  |  |  |
| 9 | Hatami et al., 2021 |  | NA |  |  |  |  |  |  |  |
| 10 | Lee et al., 2013 |  | NA |  |  |  |  |  |  |  |
| 11 | Lee et al., 2023 |  | NA |  |  |  |  |  |  |  |
| 12 | Nahum et al., 2020 |  | NA |  |  |  |  |  |  |  |
| 13 | Popova et al., 2014 |  | NA |  |  |  |  |  |  |  |
| 14 | Pot-Kolder et al., 2018 |  | NA |  |  |  |  |  |  |  |
| 15 | Priebe et al., 2015 |  |  |  |  |  |  |  |  |  |
| 16 | Siu et al., 2021 |  | NA |  |  |  |  |  |  |  |
| 17 | Subramaniam et al., 2014 |  | NA |  |  |  |  |  |  |  |
| 18 | Vass et al., 2021 |  | NA |  |  |  |  |  |  |  |
| 19 | Zhu et al., 2020 |  | NA |  |  |  |  |  |  |  |

Domain 1a: Bias arising from the randomization process. Domain 1b: Bias arising from the timing of identification or recruitment of participants (only present in Risk of Bias tool for cluster-randomized parallel-group trials). Domain 2: Bias due to deviations from intended interventions. Domain 3: Bias due to missing outcome data. Domain 4: Bias in measurement of the outcome. Domain 5: Bias in the selection of the reported result.
