# Supplementary material for: Dose-response relationship in digital psychological therapies for people with psychosis: a systematic review, meta-analysis, and meta-regression
Source: Front Psychiatry. 2025 Sep 26;16:1621009. doi: 10.3389/fpsyt.2025.1621009 (PMC12512042; doi:10.3389/fpsyt.2025.1621009)
Supplement: Supplementary file 1 [file DataSheet1.zip › Supplementary File 7.DOCX]

**Supplementary File 7 - Summary statistics for intervention and control groups at baseline and post-intervention**

| Author | Outcome | Symptom domain | Psychotherapy Group Baseline Mean (SD) | Psychotherapy Group Post-Intervention Mean (SD) | Control Group Baseline Mean (SD) | Control Group Post-Intervention Mean (SD) | Mean symptom severity change post-intervention |
| --- | --- | --- | --- | --- | --- | --- | --- |
| Bellucci et al. 2002 (32) | SANS | Negative symptoms | 13.7 (3.60) | 12.40 (3.20) | 13.10 (3.80) | 14.40 (3.80) | Intervention: decreased 1.3 points  Control: increased 1.3 points |
| Bryce et al., 2018 (33) | PANSS-T | Total psychosis symptom severity | 61.82 (3.20) | 60.96 (15.01) | 60.29 (3.28) | 57.66 (3.33) | Intervention: decreased 0.86 points  Control: decreased 2.63 points |
| Byrne et al., 2013 (34) | PANSS-T | Total psychosis symptom severity | 70.43 (22.15) | 58.21 (16.21) | 64.67 (11.41) | 62.29 (12.17) | Intervention: decreased 12.22 points  Control: decreased 2.38 points |
| Depp et al., 2018 (35) | BPRS-T | Total psychiatric symptom severity | 43.20 (9.70) | 39.70 (11.50) | 42.00 (10.90) | 41.00 (11.40) | Intervention: decreased 3.5 points  Control: decreased 1 point |
| du Sert et al., 2018 (36) | PSYRATS-AH | Positive symptoms (auditory hallucinations) | 30.47 (3.18) | 23.33 (8.44) | 30.86 (4.67) | 31.14 (2.97) | Intervention: decreased 7.14 points  Control: increased 0.28 points |
| du Sert et al., 2018 (36) | BAVQ-R | Positive symptoms (auditory hallucinations) | 22.47 (4.60) | 16.36 (7.47) | 24.29 (7.91) | 22.47 (5.41) | Intervention: decreased 6.11 points  Control: decreased 1.82 |
| Freeman et al., 2022 (6) | O-AS-A | Other symptoms (avoidance) | 3.20 (2.50) | 1.90 (2.20) | 3.40 (2.70) | 2.50 (2.60) | Intervention: decreased 1.3  Control: decreased 0.9 |
| Freeman et al., 2022 (6) | O-AS-D | Other symptoms (distress) | 51.40 (16.40) | 41.30 (18.80) | 52.60 (17.20) | 45.8 (20.4) | Intervention: decreased 10.1 points  Control: decreased 6.8 points |
| Garety et al., 2021 (37) | GPTS-T | Positive symptoms (paranoia) | 104.70 (27.60) | 84.80 (30.80) | 105.90 (26.00) | 92.5 (33.00) | Intervention: decreased 19.9 points  Control: decreased 13.4 points |
| Gottlieb et al., 2017 (38) | PSYRATS-AH | Positive symptoms (auditory hallucinations) | 38.16 (8.22) | 37.40 (6.74) | 39.83 (7.27) | 33.47 (14.01) | Intervention: decreased 0.76 points  Control: decreased 6.36 points |
| Gottlieb et al., 2017 (38) | BPRS-AH | Positive symptoms (auditory hallucinations) | 5.79 (0.97) | 5.33 (1.23) | 5.83 (1.04) | 5.00 (2.17) | Intervention: decreased 0.46 points  Control: decreased 0.83 points |
| Hatami et al., 2021 (39) | PANSS-T | Total psychosis symptom severity | 45.20 (3.80) | 42.70 (5.40) | 45.9 (3.80) | 44.20 (5.10) | Intervention: decreased 2.5 points  Control: decreased 1.7 points |
| Lee et al., 2013 (40) | PANSS-T | Total psychosis symptom severity | 63.93 (12.19) | 64.96 (13.72) | 64.11 (12.45) | 64.60 (14.08) | Intervention: increased 1.03 points  Control: increased 0.49 points |
| Lee et al., 2023 (41) | PANSS-T | Total psychosis symptom severity | 48.68 (11.44) | 43.03 (9.81) | 47.93 (9.55) | 43.33 (10.16) | Intervention: decreased 5.65 points  Control: decreased 4.6 points |
| Lee et al., 2023 (41) | PSYRATS-AH | Positive symptoms (auditory hallucinations) | 9.31 (11.19) | 8.12 (11.07) | 3.08 (6.56) | 1.84 (5.06) | Intervention: decreased 1.19 points  Control: decreased 1.24 points |
| Lee et al., 2023 (41) | PSYRATS-D | Positive symptoms (delusions) | 5.52 (7.21) | 4.33 (6.02) | 4.61 (5.67) | 4.11 (5.43) | Intervention: decreased 1.19 points  Control: decreased 0.5 points |
| Nahum et al., 2020 (42) | PANSS-T | Total psychosis symptom severity | 62.16 (15.02) | 56.10 (14.41) | 61.27 (15.55) | 55.21 (12.94) | Intervention: decreased 6.06 points  Control: decreased 6.06 points |
| Popova et al., 2014 (43) | PANSS-T | Total psychosis symptom severity | 72.20 (16.57) | 65.50 (15.62) | 69.40 (16.47) | 66.30 (18.18) | Intervention: decreased 6.7 points  Control: decreased 3.1 points |
| Popova et al., 2014 (43) | PANSS-T | Total psychosis symptom severity | 69.00 (14.06) | 60.50 (14.86) | 69.40 (16.47) | 66.30 (18.18) | Intervention: decreased 8.5 points  Control: decreased 3.1 points |
| Pot-Kolder et al., 2018 (44) | ESM-MP | Positive symptoms (paranoia) | 3.06 (1.39) | 2.71 (1.38) | 3.14 (1.43) | 3.30 (1.60) | Intervention: decreased 0.35 points  Control: increased 0.16 |
| Pot-Kolder et al., 2018 (44) | ESM-PST | Positive symptoms (perceived social threat) | 2.70 (0.86) | 2.81 (1.01) | 2.82 (0.91) | 2.84 (0.98) | Intervention: increased 0.11 points  Control: increased 0.02 points |
| Priebe et al., 2015 (45) | PANSS-T | Total psychosis symptom severity | 64.80 (16.71) | 56.30 (16.63) | 67.70 (19.93) | 62.9 (16.99) | Intervention: decreased 8.5 points  Control: decreased 4.8 points |
| Siu et al., 2021 (46) | PANSS-T | Total psychosis symptom severity | 26.41 (6.26) | 23.41 (4.88) | 27.23 (8.12) | 22.95 (3.39) | Intervention: decreased 3 points  Control: decreased 4.28 points |
| Subramaniam et al., 2014 (47) | PANSS-T | Total psychosis symptom severity | 54.28 (17.06) | 52.92 (15.56) | 53.23 (17.32) | 51.55 (15.73) | Intervention: decreased 1.36 points  Control: decreased 1.68 points |
| Vass et al., 2021 (48) | PANSS-T | Total psychosis symptom severity | 55.60 (17.51) | 58.30 (38.86) | 59.9 (19.79) | 61.30 (20.05) | Intervention: increased 2.7 points  Control: increased 1.4 points |
| Zhu et al., 2020 (49) | PANSS-T | Total psychosis symptom severity | 48.65 (10.19) | 43.14 (8.78) | 50.57 (12.5) | 47.32 (15.01) | Intervention: decreased 5.51 points  Control: decreased 3.25 points |

Outcomes: BAVQ-R - Beliefs About Voices Questionnaire – Revised; BPRS- AH - Brief Psychiatric Rating Scale Auditory Hallucinations; BPRS-T - Brief Psychiatric Rating Scale - Total; ESM-MP / PST - Experience Sampling Method - Momentary paranoia / Perceived social threat; GPTS-T - Green Paranoid Thoughts Scale - Total; O-AS-A / D - Oxford Agoraphobic Scale Avoidance / Distress; PANSS-T - Positive and Negative Syndrome Scale - Total; PSYRATS-AH / D - Psychotic Symptom Rating Scales Auditory Hallucinations / Delusions; SANS – Scale for the Assessment of Negative Symptoms.
